# Comparison of three common shoulder injections for rotator cuff tears: a systematic review and network meta-analysis

**DOI:** 10.1186/s13018-023-03747-z

**Published:** 2023-04-03

**Authors:** Xinzhao Jiang, Hong Zhang, Qing Wu, Yun Chen, Tian Jiang

**Affiliations:** 1 Department of Orthopedics, The First Affiliated Hospital of Soochow University, Suzhou, Jiangsu China; 2 Department of Pain Management, Jiangyin People’s Hospital Affiliated to Nantong University, Wuxi, 214400 China

**Keywords:** Rotator cuff tear, Network meta-analysis, Platelet-rich plasma, Hyaluronic acid, Corticosteroid

## Abstract

**Objective:**

To compare the clinical effectiveness of three common shoulder injections mentioned in the guidelines [corticosteroid, sodium hyaluronate (SH) and platelet-rich plasma (PRP)] on rotator cuff tears.

**Material and methods:**

The PubMed, Embase and Cochrane Library databases were systematically searched up to June 1, 2022, for randomized controlled trials (RCTs) and prospective studies on the three injection therapies for rotator cuff tears. The main results were pain relief and functional improvement at 1–5 months and over 6 months, pooled using a network meta-analysis and ranked by SUCRA score. The risk of bias of the included studies was assessed using the Cochrane Collaboration tool.

**Results:**

Twelve RCTs and 4 prospective studies comprising a total of 1115 patients were included in the review. Three prospective studies were judged to be at high risk of selection bias and performance bias, and one was considered as having a high risk of detection bias. SH injection ranked first in the short term in pain relief (MD: − 2.80; 95%CI − 3.91, − 1.68) and functional improvement (MD:19.17; 95%CI 12.29, 26.05), while PRP injection obtained better results in the long term in both pain relief (MD: − 4.50; 95%CI − 4.97, − 4.03) and functional improvement (MD:11.11; 95%CI 0.53,21.68).

**Conclusions:**

PRP injection has the potential to successfully treat rotator cuff tears as an alternative to corticosteroids in the long term, in terms of either therapeutic efficiency or adverse effects, followed by SH injection. More research is needed to make high-quality recommendations on treatment options for injection treatments of rotator cuff tears.

**Supplementary Information:**

The online version contains supplementary material available at 10.1186/s13018-023-03747-z.

## Introduction

Rotator cuff tears are a common musculoskeletal disorder and a major cause of shoulder pain, with injury and degeneration being the two main causes [1]. The prevalence of rotator cuff tears increases with age, from 9.7% in patients aged 20 years and younger to 62% in patients aged 80 years and older [2]. A recent study found that patients with rotator cuff tears returned to previous work at approximately 8 months after surgery, and more than 35% of them could not return to their previous level of work [3].

Treatments for rotator cuff tears range from noninvasive physical therapy to more invasive procedures such as shoulder injections and surgery. There is no clear consensus on the best way to treat patients with rotator cuff tears so far. Although surgical repair is the standard treatment for rotator cuff tears, the poor self-repair capability of the tendon leading to a high retear rate and the financial and time pressures on patients make conservative therapies equally important [4]. Conservative treatment consists of several interventions, including physiotherapy like scheduled stretching and strengthening exercises, systemic medications such as pain medication and anti-inflammatory drugs, intraarticular injections and hyperthermia [5, 6]. Clinical practice guidelines for rotator cuff injuries have mentioned three injection therapies [corticosteroid, platelet-rich plasma (PRP) and hyaluronic acid (HA)] were mentioned for the nonsurgical management of patients; however, the guideline strength of recommendation for PRP and HA injections was limited, and corticosteroids were moderate, although a large number of studies and meta-analyses on injection therapies have been performed [7–11]. Intraarticular steroid injections reduce aseptic inflammation of the synovium, shoulder capsule and surrounding tissues. The PRP injection collects the patient’s own plasma and injects it into the joint capsule to help revascularize the torn part area and promote tissue recovering. Hyaluronic acid could not only lubricate the shoulder joint but also suppress the inflammatory response. The previous systematic reviews show different views on the effectiveness of these three injections. Some studies suggest that PRP injections may have a positive effect on clinical outcomes such as pain relief and long-term retear rates [9, 10]. However, another meta-analysis found no statistically significant differences between PRP and other conservative treatments [8]. Meanwhile, a study on PRP injection considered it as not cost-effective on rotator cuff tears despite the reduced retear rate [4]. Similarly, there is controversy over the short- and long-term effects of corticosteroids and hyaluronic acid [12, 13]. The varied classification of patients and treatments may lead to the bias.

Most of the current studies focus on comparisons between single drug injections, and direct comparisons between multiple drug injection categories are lacking. A better understanding of the comparative efficacy of these therapies is expected to help physicians refine treatment strategies for rotator cuff tears. A network meta-analysis can make up for the deficiency of traditional meta-analysis and compare of multiple treatments simultaneously by integrating both direct and indirect evidence [14]. Therefore, the aim of the present analysis is to evaluate the clinical effects of these three injection therapies on patients with rotator cuff tears and give a ranking according to their short-term and long-term effects for practical application.

## Methods

The detailed protocol for this study was designed according to the Cochrane intervention review and has been registered on the PROSPERO website (CRD42022336258).

### Search strategy

This review was conducted according to the standards of the Preferred Reporting Items for Systematic Reviews and Meta-Analysis (PRISMA) guidelines [15]. We used a combination of keywords, Medical Subject Headings and entry terms to conduct an extensive literature search on PubMed, Embase and the Cochrane Library in June 1, 2022. Web of Science and Scopus were not within the scope of our literature search. The search strategy is available in Additional file 1. The gray literature, including books and conference proceedings, was searched via the Opengrey database (https://opengrey.eu/) and Google Academic; meanwhile, we manually checked the latest review or similar meta-analysis related our study to obtain the documents that may be missed during the retrieval process. The search had no language restrictions, and the search period was from June 1, 2003, to June 1, 2022 (last 20 years).

### Eligibility criteria

We constructed eligibility criteria using the population, intervention, control/comparison and outcome models (PICO). (1) Participants: We included adults (> 18 years of age) of either sex diagnosed with any type of degenerative, traumatic, partial or full-thickness rotator cuff tears confirmed by clinical symptoms, medical history, physical examination and imaging evaluation (ultrasound, magnetic resonance imaging (MRI) or arthrography). The definition of rotator cuff tears was derived from the guidelines and previous reviews on rotator cuff injuries [7, 16]. Trials that only include patients with shoulder pain, calcific tendinitis or subacromial impingement syndrome were excluded from our studies unless they also included patients with any type of rotator cuff tears. (2) Intervention and Comparison: Trials treated with at least 2 arms of nonoperative injection therapies, including corticosteroid, PRP, SH and placebo, were eligible. (3) Outcomes: The primary measures of treatment effect were pain reduction and improvement in shoulder function, including the Visual Analog Scale (VAS), Constant–Murley scores (Constant), Western Ontario Rotator Cuff Index (WORC) and American Shoulder and Elbow Surgeons Standardized Form (ASES). (4) Randomized controlled trials (RCT) and prospective studies were included in our review, and literature reviews, expert consensus, nonclinical studies or case reports were all excluded.

### Data collection and quality assessment

Two authors independently screened the full text and extracted all the data, including the baseline demographic characteristics, symptoms, injection dosage, injection site, outcome measures, adverse effects and the time points of follow-up assessments. Disagreements between the results were resolved through a third independent author. The outcomes calculated in the meta-analysis were the VAS pain score and the constant score. Outcomes were extracted separately for the short and long term, with an assessment at a time point of less than 6 months being defined as a short-term effect and more than 6 months being defined as a long-term effect. Two reviewers independently performed a quality assessment of the trials. The Cochrane Collaboration tool was used to evaluate the risk of bias as high, low or unclear, which covers the following domains: random sequence generation, allocation concealment, blinding, incomplete outcome data, selective reporting and other bias. The quality of evidence from the network meta-analysis for each network contrast was estimated in terms of the Grading of Recommendations, Assessment, Development and Evaluations (GRADE) framework, which could be rated from high, moderate, low to very low.

### Statistical and inconsistency analysis

The network meta-analysis was performed using the “network” package in Stata (version 15.0). Comparisons between different therapies are presented using network plots, where the size of the nodes represents the total sample size of multiple treatments and the width of the lines represents the number of studies between 2 treatments. We used the mean difference (MD) and 95% credible interval (CI) to compare the outcome change between 2 different injection therapies, using the frequentist approach to random-effects network meta-analysis. The Wald test and node-splitting analysis were adopted to evaluate the overall and local inconsistencies within the network, respectively, and the consistency model was used to calculate the pooled effect size if the p value of the inconsistency analysis was more than 0.05. The surface under the cumulative ranking curve (SUCRA) was used to calculate the probabilities of each treatment being the best among all therapies. Publication bias was assessed using funnel plots.

## Results

### Search results

**Fig. 1:**
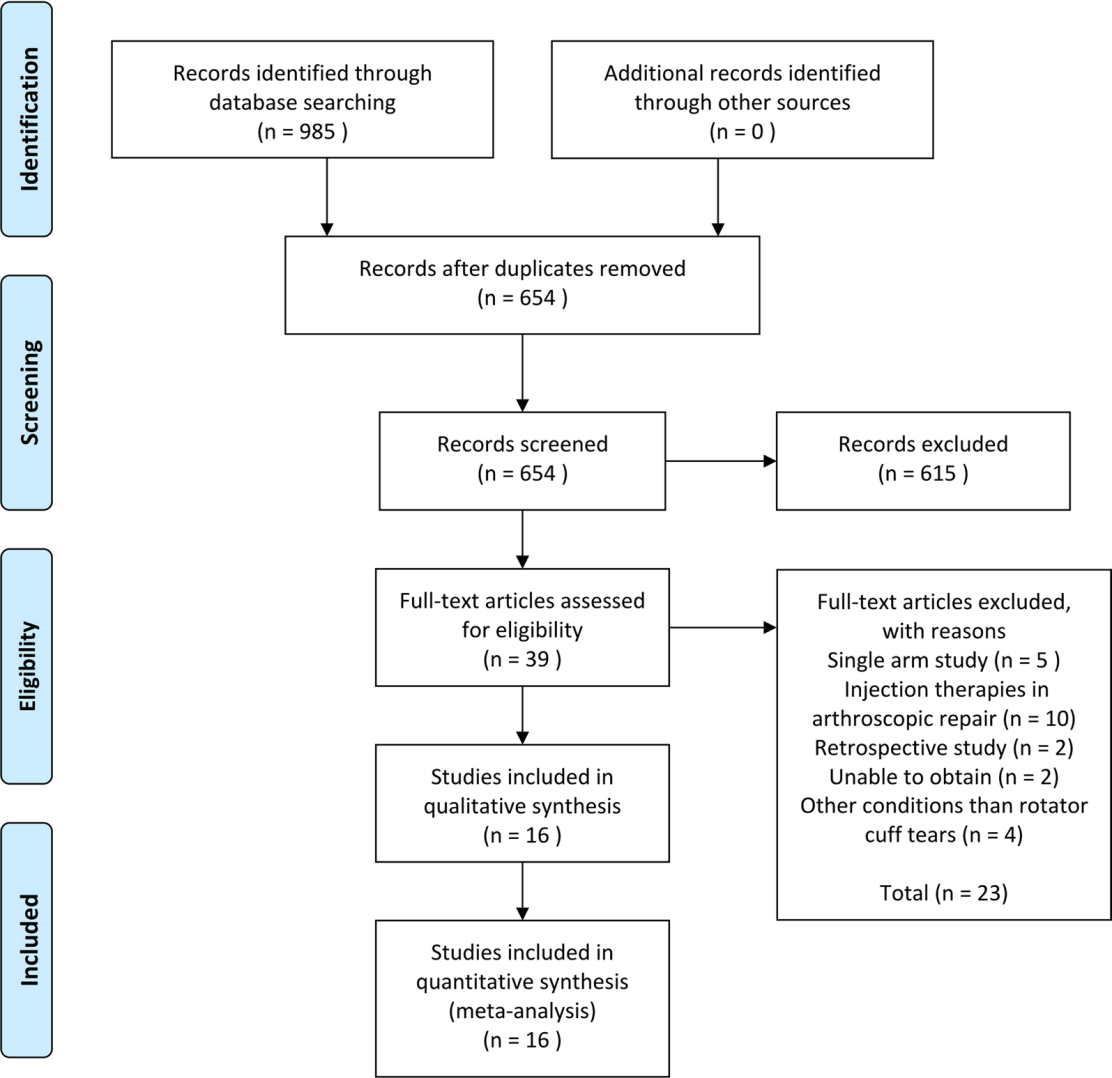
Flow diagram of the study selection process

A total of 985 articles were retrieved from the initial search of the major databases, and 654 articles remained after duplicate articles were excluded. We discarded 615 articles by screening the titles and abstracts, and 16 studies were finally included in this review after evaluation of the full-text articles, including 12 RCTs [17–28] and 4 prospective studies [29–32] with a total of 1115 patients (Fig. 1). Two studies [18, 26] included 4 types of interventions, and 2 studies [21, 30] adopted 3 types of interventions. Only one study included patients receiving conservative therapies with full-thickness rotator cuff tears [21], while the others included participants with partial rotator cuff tears. The average age of the patients in the trials ranged from 39.0 to 79.4 years. Among them, 16 studies reported short-term outcomes (1–5 months), and 11 studies reported long-term outcomes (over 6 months). Regarding the location of injection, 14 studies performed subacromial injections, and 2 performed intraarticular injections. The characteristics of the included studies are available in Table 1.

**Table 1:** Characteristics of included studies

Reference	Study type	Interventions	Inclusion criteria	Number of patients	Age	Gender (male/female)	Dropout Rate	Injection	Outcome measure	Follow-up	Adverse effects	Main results
Byun et al. [17], Korea	RCT	Corticosteroid vs. corticosteroid + SH	Partial or full-thickness tear of rotator cuff or subacromial bursitis	13/13	55.5/55.4	6/20	NR	1 mL of triamcinolone acetonide (40 mg) and 5 mL of 0.5% lidocaine with or without SH; Subacromial injection	ROM, VAS, SFA, SDQ	3w	NR	The combination injection of corticosteroid had additive effects on the improvement of AROM and SFA scores
Cai et al. [18], China	RCT	SH vs. PRP vs. SH + PRP vs. placebo	Partial rotator cuff tears	44/45/48/47	39/41/40/40	99/85	7/184	SH: 4 mL PRP: 4 mL SH + PRP: 2 mL of SH and 2 mL of PRP Placebo: 4 mL of normal saline; Subacromial injection	ASES, VAS, constant score	1 m, 3 m, 6 m, 12 m	NR	The SH + PRP group showed a significantly improvement in all outcomes at 12 months compared with SH or PRP alone
Chou et al. [19] Taiwan	RCT	SH vs. placebo	Rotator cuff tears without complete tears	25/26	51.1/52.4	19/32	NR	25 mg of SH or 2.5 mL of normal saline; Subacromial injection	Constant score, VAS, global improvement	6w	NR	The SH group had a better Constant score(p < 0.0095) and VAS score (p < 0.0018) than the placebo group 6 weeks after treatment
Dadgostar et al. [20], Iran	RCT	PRP vs. corticosteroid	Incomplete rotator cuff tear or tendinitis	30/28	57.3/53.6	11/47	NR	3 mL of PRP or 1 mL of Depo-medrol (40 mg) and 1 mL of lidocaine (2%); Subacromial injection	ROM, VAS, WORC, DASH	1w, 1 m, 3 m	NR	The PRP group had significant improvements in ROM and VAS during 3 months follow-up
Gialanella et al. [21], Italy	RCT	Corticosteroid (single injection) vs. corticosteroid (two injections) vs. placebo	Full-thickness rotator cuff tear	20/20/20	78.7/77.3/79.4	5/55	NR	G1: single intraarticular injection of 40 mg triamcinolone acetonide G2: two injections of 40 mg triamcinolone acetonide G3: no treatment; Intraarticular injection	VAS, Constant score	1 m, 3 m, 6 m	NR	Corticosteroid improved VAS scores at 1 and 3 months (p < 0.05), but two injections of steroids did not enhance the effect
Huang et al. [29], Taiwan	Prospective	PRP (single injection) vs. HA (three injections)	Partial rotator cuff tears	24/24	61.8/61.8	11/37	6/54	4 mL of PRP or 2 mL of HA; Subacromial injection	SPADI, constant score, VAS, ROM	1 m, 3 m	NR	Single PRP injection exhibited comparable benefit to three doses of HA injection short-termly
Jo et al. [22], Korea	RCT	PRP vs. corticosteroid	Partial rotator cuff tears or tendinopathy	30/30	55.3/52.5	20/40	11/60	4 mL of allogeneic PRP or a 4 mL mixture of 1 mL of 40 mg/mL triamcinolone acetonide and 3 mL of 2% lidocaine; Subacromial injection	Constant score, VAS, ROM, SPADI, ASES, SST, DASH	1w, 1 m, 3 m, 6 m	NR	No significant difference were reported between two groups in constant score at 1 month, but the PRP group had better DASH score, overall function and external rotation than steroid group
Kesikburun et al. [23], Turkey	RCT	PRP vs. placebo	Rotator cuff tendinosis or partial tendon tear	20/20	45.5/51.4	13/27	NR	5 mL of PRP or 5 mL of normal saline; Injection under the posterolateral aspect of the acromion; Subacromial injection	WORC, SPADI, VAS, ROM	3w, 6w, 12w, 24w, 1y	NR	PRP injection was found to be no more effective in WORC, SPADI and VAS scores than placebo in patients treated with an exercise program
Kwong et al. [24], Canada	RCT	PRP vs. corticosteroid	Rotator cuff tendinopathy or partial tears	47/52	49.9/49.1	35/64	NR	5 mL of PRP or a mixture of 1 mL of 40 mg/mL triamcinolone and 2 mL of 0.5% bupivacaine; Subacromial injection	VAS, ASES, WORC	6w, 3 m, 12 m	NR	PRP group obtained more improvement in pain and function at 3 months follow-up, but no sustained benefit at 12 months follow-up
Moghtaderi et al. [25], Iran	RCT	SH vs. placebo	Rotator cuff disease without complete tears	20/20	NR	14/26	NR	2 mL of SH (20 mg) or 2 mL of normal saline; Subacromial injection	Constant score, VAS	1w, 2w, 3w, 12w	NR	The SH group had better improvements in VAS and constant score compared with placebo
Sari et al. [26], Turkey	RCT	PRP vs. corticosteroid vs. prolotherapy vs. placebo	Rotator cuff tendinosis or partial tears	33/33/32/31	52.1	43/77	9/129	G1: 5 mL of PRP G2: 2 mL 40 mg triamcinolone acetonide and 1 mL 1% lidocaine and 1 mL saline G3:a mixture of 4 mL 20% dextrose and 1 mL lidocaine G4: a mixture of 3 mL 1% lidocaine 134 and 2 mL saline solution; subacromial injection	VAS, ASES, WORC	3w, 12w, 24w	NR	The steroid group had lower VAS and WORC scores than other groups in the 3^rd^ week. In the PRP group in the 24^th^ week, VAS and WORC scores were found to be significantly lower than the COR group
Schwitzguebel et al. [27], Switzerland	RCT	PRP vs. placebo	Interstitial supraspinatus tears	41/39	48.2/47.6	45/80	4/84	2 mL of PRP or 2 mL of normal saline; Subacromial injection	VAS, ASES, constant score, SANE	7 m, 12 m	The PRP group had significantly higher incidence of adverse effects (pain 48 h, frozen shoulder, extension of lesion to bursal or articular surface) compared with the control group (54% vs 26%)	PRP injections tears did not improve tendon healing or clinical scores compared with saline injections and were associated with more adverse events
Setaro et al. [30], Italy	Prospective	Corticosteroid vs. HA (median molecular weight) vs. HA (high molecular weight)	Partial rotator cuff tears	20/20/20	57	26/34	NR	G1: 40 mg of methylprednisolone acetate G2: 40 mg of medium molecular weight HA (1000–1500 kDa) G3: 40 mg of high molecular weight HA (2500–3500 kDa); Intraarticular injections	VAS, OSS, constant score, ROM	2w, 1 m, 2 m, 4 m	NR	Medium molecular weight HA showed greater efficacy in terms of pain reduction and functional recovery of the shoulder joint
Shams et al. [28], Egypt	RCT	PRP vs. corticosteroid	Partial rotator cuff tears	20/20	51	21/19	NR	5 mL of PRP or 5 mL of triamcinolone acetonide (40 mg); Subacromial injection	Constant score, ASES, SST, VAS	6w, 12w, 6 m	NR	The PRP group showed better results as compared to corticosteroid injections at 12 weeks, although statistically significant better results after 6 months could not be found
Tagliafico et al. [31], Italy	Prospective	HA vs. control	Massive rotator cuff tears	30/60	72/71	36/54	NR	Subacromial injection of HA (500 to 730 kD)	Constant score, VAS	1 m, 2 m, 3 m, 4 m, 5 m, 6 m	NR	The HA group presented a significant improvement in VAS and constant scores at 1–4 months, but no difference after 5 months follow-up
Wehren et al. [32], Switzerland	Prospective	PRP vs. corticosteroid	Partial rotator cuff tears	25/25	53/55	26/24	NR	5 mL of PRP or 5 mL of triamcinolone acetonide (40 mg); Subacromial injection	Constant score, ASES, SST, VAS	6w, 12w, 6 m	NR	PRP injections show earlier benefit as compared to corticosteroid injections at 6 and 12 weeks, although a statistically significant difference after 6 months could not be found

ASES, American Shoulder and Elbow Surgeons Standardized Form; DASH, Disabilities of the Arm, Shoulder and Hand; NR, not reported; OSS, Oxford shoulder score; PRP, platelet-rich plasma; ROM, range of motion; SANE, Single Assessment Numerical Evaluation; SDQ, Shoulder Disability Questionnaire; SFA, Shoulder Function Assessment Scale; SH, sodium hyaluronate; SPADI, Shoulder Pain And Disability Index; SST, Simple Shoulder Test; UCLA, University of California at Los Angeles scores; VAS, Visual Analog Scale Scores; WORC, Western Ontario Rotator Cuff Index

### Quality assessment

**Fig. 2:**
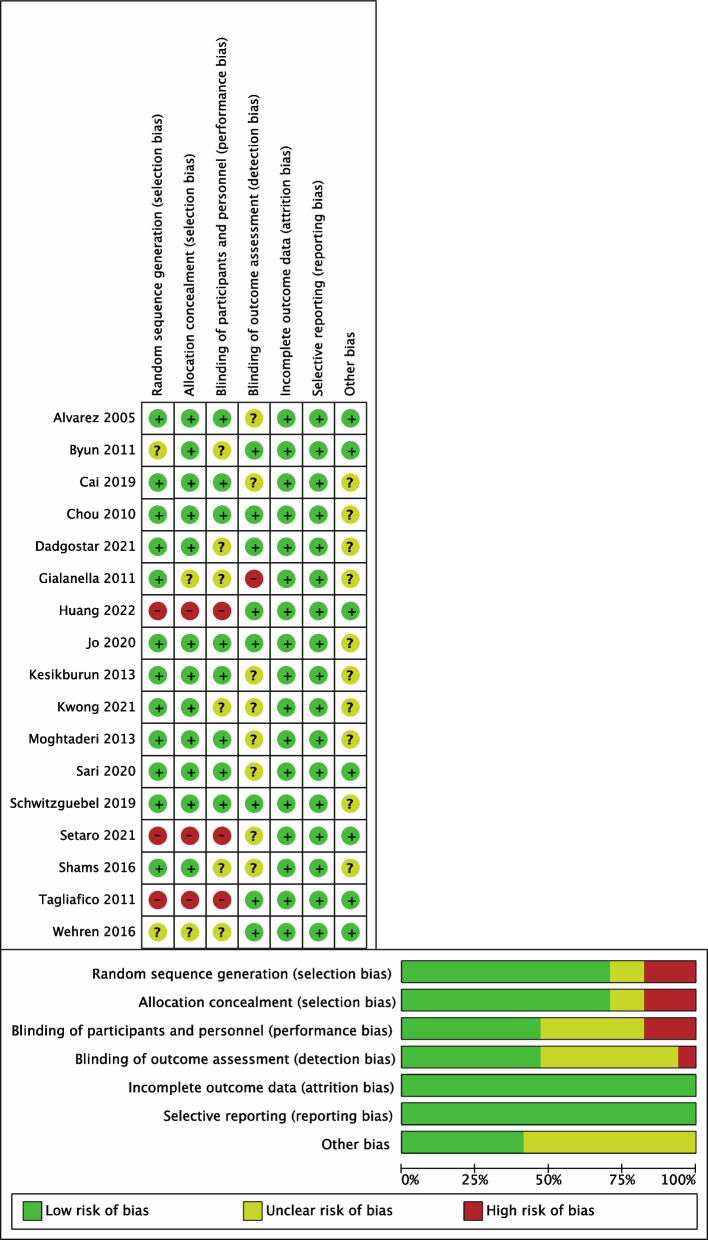
Risk of bias assessment for all included studies

The risk of bias summary and graph are presented in Fig. 2. Three prospective studies were judged to be at high risk of selection bias and performance bias because they generated the treatment allocation schedule according to the patients’ wishes, resulting in a lack of blinding of the patients and personnel [29–31]. We rated the study by Gialanella et al. as having a high risk of detection bias because the outcomes were measured by the same physician who performed the injection therapies [21]. All the studies were assessed as having a low risk of incomplete outcome data for the minor and balanced loss to follow-up between groups.

### Network geometry

**Fig. 3:**
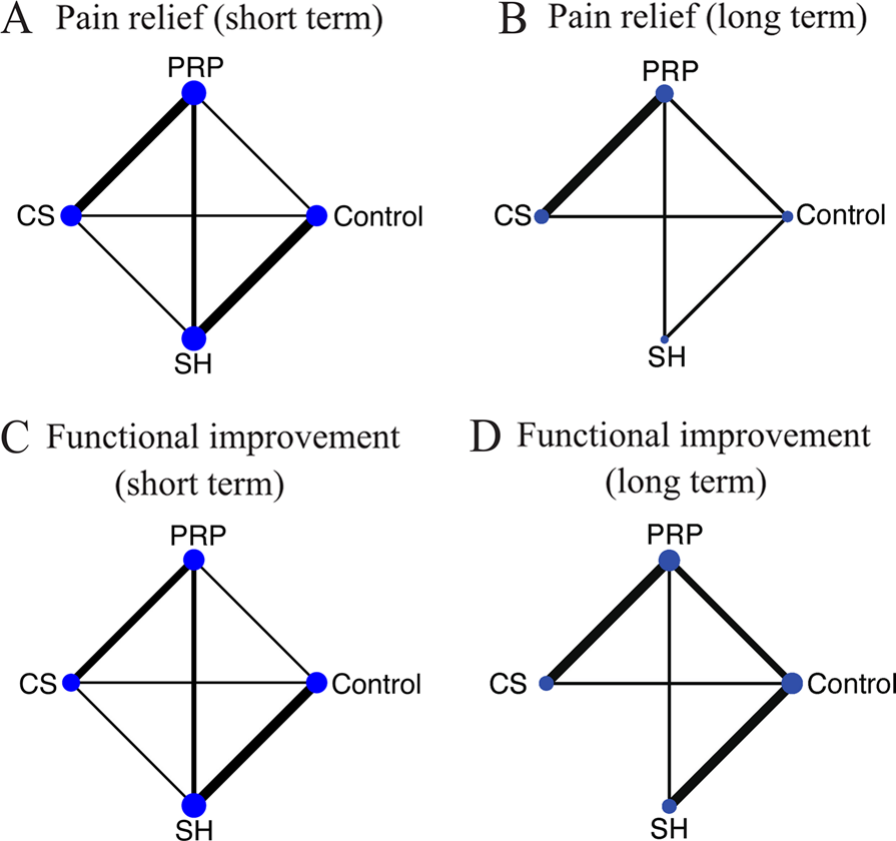
Network map of the studies included in the network meta-analysis: **A** pain relief in short-term follow-up, **B** pain relief in long-term follow-up, **C** functional improvement in short-term follow-up, **D** functional improvement in long-term follow-up. CS, Corticosteroid; PRP, platelet-rich plasma; SH, sodium hyaluronate

The comparison network plot for pain relief and functional improvement in the short/long term is presented in Fig. 3. All three interventions were directly compared with controls. The short-term results for VAS and constant score showed closed loops, while direct comparisons between corticosteroid and SH for the long-term outcomes were lacking.

### Inconsistency analysis

The results of the inconsistency analysis are available in Table 2. The node-splitting analysis detected inconsistency only in the short-term VAS score of the comparison between PRP and control groups (*p* = 0.009), and the Wald test reported no significant global inconsistency in these loops (*p* > 0.05). Thus, we used the consistency type to perform the network meta-analysis.

**Table 2:** Results of the global and local inconsistency

	VAS score (short term)	VAS score (long term)	Constant score (short term)	Constant score (long term)
Control versus PRP	0.009	0.143	0.291	0.903
Control versus CS	0.306	0.144	0.646	0.994
Control versus SH	0.765	0.143	0.375	0.050
PRP versus CS	0.447	0.144	0.422	0.995
PRP versus SH	0.692	0.143	0.106	0.436
CS versus SH	0.605	NA	0.686	NA
Global inconsistency	0.179	0.144	0.450	0.107

### Effectiveness of the inventions

**Fig. 4:**
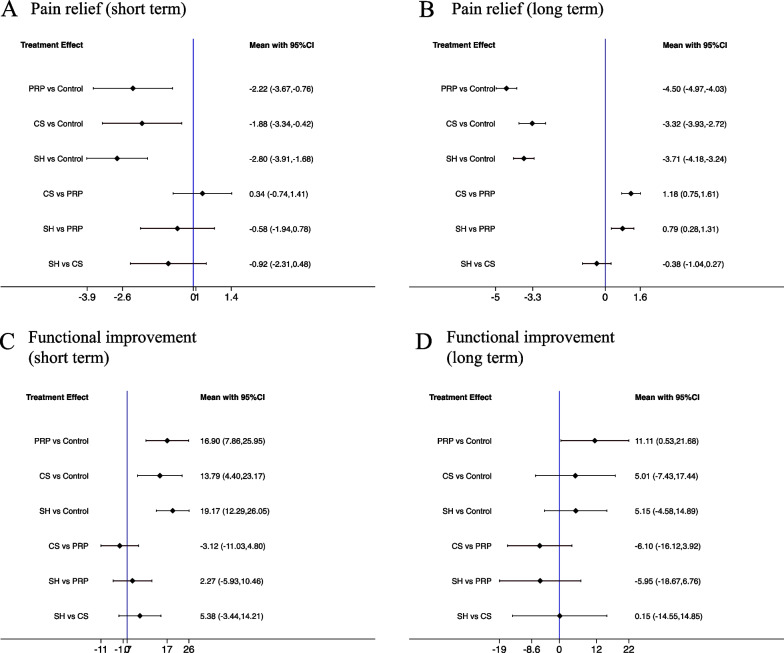
Forest plot of the network meta-analysis: comparison of the three treatments in pain relief and functional improvement in the short/long term: **A** pain relief in short-term follow-up, **B** pain relief in long-term follow-up, **C** functional improvement in short-term follow-up, **D** functional improvement in long-term follow-up. CS, Corticosteroid; PRP, platelet-rich plasma; SH, sodium hyaluronate

The results of the network meta-analysis are shown in Fig. 4. A total of 11 trials with 775 patients were included in the analysis for short-term pain relief [18–22, 24–26, 29–31]. The extent of pain relief was evaluated by the change in the VAS score, which ranged from 0 to 10, with lower MD values indicating better effectiveness. The pooled network MD values indicated that all three interventions (CS, PRP, SH) showed significant superiority over the control group in terms of pain relief, with SH therapy leading to a greater reduction in the VAS score (MD: − 2.80; 95% CI − 3.91, − 1.68) (moderate certainty evidence). There is no significant difference in the comparisons between these three interventions. The analysis for long-term pain relief contained 5 studies with 395 patients [18, 21, 22, 24, 26]. The long-term efficacy of these three therapies compared with the control groups was better than that in the short term, and PRP injection had the greatest reduction in VAS score (MD: − 4.50; 95% CI − 4.97, − 4.03). PRP was also reported to have better improvement in pain relief than CS (MD: − 1.18; 95% CI − 1.61, − 0.75) and SH (MD: − 0.79; 95% CI − 1.31, − 0.28) in the long term (moderate certainty evidence).

Ten trials with 648 patients provided short-term data on functional improvement of the shoulder [18, 19, 21, 22, 25, 28–32], and a constant score ranging of 0–100 was used to assess this outcome, with higher MD values indicating better efficacy. All three treatments showed superiority over control groups in short-term functional improvement, with SH obtaining greater improvement in the constant score (MD: 19.17; 95% CI 12.29, 26.05) (moderate certainty evidence). A total of 8 studies with 589 patients reported a constant score at long-term follow-up [18, 21, 22, 25, 27, 28, 31, 32], and the pooled result revealed that only PRP therapy had a statistically significant benefit over the control group (MD: 11.11; 95% CI 0.53, 21.68) (moderate certainty evidence).

**Fig. 5:**
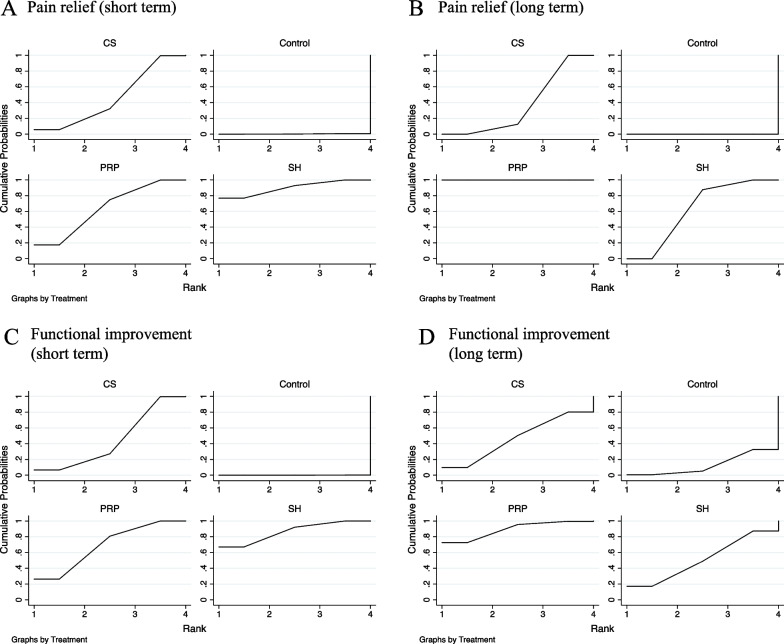
Rank probability for the three treatments in pain relief and functional improvement in the short/long term: **A** pain relief in short-term follow-up, **B** pain relief in long-term follow-up, **C** functional improvement in short-term follow-up, **D** functional improvement in long-term follow-up. CS, Corticosteroid; PRP, platelet-rich plasma; SH, sodium hyaluronate

The SUCRA analysis provided a ranking of these three injection therapies according to their efficacy in improving the VAS and constant score (Fig. 5). According to the ranking results shown in Table 3, SH therapy ranked first at short-term follow-up and might be the best injection treatment in terms of pain relief (SUCRA score: 89.9) and functional improvement (SUCRA score: 86.4). Nevertheless, PRP injection seemed to be the best injection treatment in both pain relief (SUCRA score: 100.0) and functional improvement (SUCRA score: 89.2) in the long term. The funnel plots are presented in Additional file 2, which showed a possible low risk of publication bias in functional improvement.

**Table 3:** Results of the SUCRA score

Treatment	VAS score (short term)	VAS score (long term)	Constant score (short term)	Constant score (long term)
Control	0.3	0.0	0.1	12.9
PRP	64.0	100.0	69.0	89.2
CS	45.8	37.5	6.6	46.8
SH	89.9	62.5	86.4	51.2

### Effectiveness of the combined therapies

Two trials investigated the additive effects of the combined therapies of the three injections [17, 18]. Byun et al. made a comparison between subacromial bursa injection of hyaluronate with steroid and corticosteroid alone in patients with partial or full-thickness rotator cuff tears [17]. Both groups were found to have statistically significant improvements in VAS and shoulder disability questionnaire (SDQ) scores; however, the active range of motion (AROM) and shoulder function assessment scale (SFA) showed significant improvement only in the combination therapy group. Cai et al. evaluated the combined use of SH and PRP in the treatment of small to medium rotator cuff tears [18]. The SH + PRP group was reported to have significant improvement in the constant VAS and ASES scores compared with SH or PRP injection alone at the 12-month follow-up, along with a significant reduction in tear size from the MRI scan.

## Discussion

Our updated systematic review and network meta-analysis is a further exploration of the therapeutic effects of three shoulder injections for rotator cuff tears. Fifteen of the 16 studies included in our review focused on patients with partial thickness rotator cuff tears, for whom nonoperative treatment is a viable first-line option with a low risk of fatty infiltration, tear progression and muscle atrophy [33]. Physiotherapy, medicine injections and activity modification are common options for nonoperative rotator cuff repair; however, if the underlying tears are not addressed, over 40% of partial thickness defects would progress to full-thickness tears within three years [34]. There have been many trials and meta-analyses comparing treatments for rotator cuff tears, but very few have focused on the integration of injection treatments. Maillot et al. performed a network meta-analysis of multiple treatments for massive rotator cuff tears and found that PRP injections did not appear to provide any additional benefit [8]. This finding differs from the results of some previous meta-analyses which favored PRP injection for rotator cuff repair [9, 10]. This discrepancy might be due to the fact that Maillot et al. did not exclude studies evaluating the efficacy of PRP injection in arthroscopic repair. Lin et al. compared the effectiveness of injection therapies mainly in rotator cuff tendinopathy, including chronic tendinosis, partial cuff tears, subacromial impingement syndrome, etc. [11], and corticosteroids were found to be beneficial in the short term, whereas PRP and prolotherapy yielded better long-term outcomes. Despite their similarity to ours at long-term conclusions, the different diagnostic labels used in their inclusion criteria could lead to heterogeneity.

The present study showed better short-term improvements in pain relief and shoulder function with SH injection for patients with rotator cuff tears. Hyaluronate is a major component of the synovial fluid on the surface of articular cartilage and can act as a lubricant and shock absorber in the movements of the joint [35]. In regard to the efficacy of SH in rotator cuff tears, as mentioned in the guideline from the American Academy of Orthopedic Surgeons, there is limited evidence to support the use of SH injections in nonsurgical treatment. Osti et al. reported the function of SH in improving VAS and functional scores without serious adverse reactions in a systematic review of 11 prospective trials [36]. Frizziero et al. demonstrated the prompt clinical improvement of intraarticular HA injection on patients with rotator cuff tendinopathies and was not lost to extracorporeal shock therapy [37]. Due to the tear, the subacromial bursa can communicate with the tendon in the tear on the side of the bursa, and SH can penetrate into the tear site and surrounding tissue. SH has beneficial effects on both the repair site and the synovial sheath, participating in the repair process through epithelial and endothelial cells, reducing peripheral inflammatory responses and promoting contact healing [38, 39]. Gallorini et al. found that hyaluronic acid could improve cell escape from H_2_O_2_-induced oxidative stress and decrease cytotoxicity by reducing Nrf2 expression in human tenocytes, thereby counteracting inflammation [40]. The increased viability and proliferation of extracellular matrix cells induced by SH have also been demonstrated in some studies [41, 42].

In the present study, PRP injection showed great efficacy in both pain relief and functional improvement at long-term follow-up. PRP is obtained by centrifugation of whole blood collected from patients, resulting in a platelet-rich fraction with a higher platelet concentration than whole blood [43]. PRP was injected into the injury site to stimulate healing at the tendon–bone interface with a high concentration of platelets and growth factors, including platelet‐derived growth factor (PDGF), transforming growth factor-*β* (TGF‐*β*), fibroblast growth factor (FGF), insulin‐like growth factor (IGF‐I, IGF‐II) and vascular endothelial growth factor (VEGF) [44]. Several studies have demonstrated the potential benefit of promoting tendon matrix repair in tendon-related disorders [45]. With regard to rotator cuff disease, there are few meta-analyses that include nonsurgical cases only. Xiang et al. reported a significant effect of PRP as a conservative therapy with a constant score in both the short and long term, which is consistent with our findings, but no long-term effect on pain relief was observed. Their subgroup analysis also found that PRP with double centrifugation was associated with better recovery for the presumably higher platelet concentration compared with a single centrifugation [46, 47]. Lui et al. found that nonoperative PRP injection reduced pain from 3 to 12 months after injection but no significant improvement compared with physical therapy [48]. Other studies have focused on the effect of PRP in patients receiving ARCR. Wang et al. demonstrated that PRP injection could significantly improve the short-term outcomes after arthroscopic repair of full-thickness rotator cuff tears and reduce the retear rate with single-row fixation [49], and a meta-analysis of 13 trials by Ahmad et al. reported a similar result [50]. The analysis by Warth et al., however, found no significant differences in overall gain in outcome scores or retear rates between groups with and without PRP supplementation after rotator cuff repair [51]. This may be related to the differences in PRP preparation, such as the platelet count and leukocyte concentrations. In vitro studies have proved that leukocyte-reduced PRP promotes normal collagen matrix synthesis and reduces cytokines associated with matrix degradation and inflammation to a greater extent than high-leukocyte concentrated PRP [52]. Further studies are needed to determine the potential mechanisms and efficiency of combined therapies.

There are a few limitations that cannot be ignored in this review. Firstly, further classification of patients can be made with less than 6 months of follow-up (e.g., 1–3 months and 3–5 months) to increase the credibility of the results. Second, the treatment protocols and doses varied; for example, patients in some studies received more than one injection, while others included additional treatments such as physical exercise. Third, future studies should use a more accurate classification of patients, as the efficiency of injection therapies might vary in patients with partial, massive, incomplete rotator cuff tears, not to mention tendinopathy or subacromial bursitis. Fourth, failure to search certain databases like Web of Science or Scopus may result in the omission of articles. Fifth, the certainty of evidence in our study was mainly downgraded for study limitations, as what we have drawn in the risk of bias assessment, blinding the participants and staff was difficult sometimes for some comparisons, and unblinded outcome assessments may also be biased in effect estimates. Finally, the conceptual and statistical heterogeneity, such as different outcome measures and clinical scores used, and the inconsistency might introduce errors into our meta-analysis.

## Conclusion

The present network meta-analysis demonstrated that among the three injection treatments in patients with rotator cuff tears, SH injection plays a role in short-term [1–5] functional improvement and pain relief, while PRP injection may achieve better results in long-term follow-up (over 6 months). Corticosteroids, although one of the most common therapies, may not be as good as the above two therapies in terms of therapeutic effect and safety. However, how to get these therapies out of their maximum function is not clear, i.e., the site and numbers of injections, the dosages and whether they should be combined with other treatments. More research is needed to make high-quality recommendations on treatment options for injection treatments of rotator cuff tears.

## Supplementary Information

**Additional file 1**. Search strategy

**Additional file 2**. Funnel plot of the network meta-analysis

## Data Availability

The datasets used and/or analyzed during the current study are available from the original source articles or from the corresponding author on reasonable request.
