# Supplementary material for: Comparison of three common shoulder injections for rotator cuff tears: a systematic review and network meta-analysis
Source: J Orthop Surg Res. 2023 Apr 3;18:272. doi: 10.1186/s13018-023-03747-z (PMC10069022; doi:10.1186/s13018-023-03747-z)
Supplement: Supplementary file 1 — Additional file 1. Search strategy [file 13018_2023_3747_MOESM1_ESM.docx]

Search strategy

Search: ((((Rotator Cuff Injuries[MeSH]) OR (Rotator Cuff Injuries)) OR (Rotator Cuff Tears)) OR (Rotator Cuff Tendinosis)) AND ((((((Platelet-Rich Plasma[MeSH]) OR (Platelet-Rich Plasma)) OR (PRP)) OR (Plasma, Platelet-Rich)) OR (((Hyaluronic Acid[MeSH]) OR (Hyaluronic Acid)) OR (Sodium Hyaluronate))) OR ((((Adrenal Cortex Hormones[MeSH]) OR (Adrenal Cortex Hormones)) OR (Corticosteroids)) OR (Corticoids)))
