# Supplementary material for: Comparison of three common shoulder injections for rotator cuff tears: a systematic review and network meta-analysis
Source: J Orthop Surg Res. 2023 Apr 3;18:272. doi: 10.1186/s13018-023-03747-z (PMC10069022; doi:10.1186/s13018-023-03747-z)
Supplement: Supplementary file 2 — Additional file 2. Funnel plot of the network meta-analysis [file 13018_2023_3747_MOESM2_ESM.docx]

**Supplement 2** Funnel plot of the network meta-analysis

(A)Pain relief in short-term follow-up, (B)Pain relief in long-term follow-up, (C)Functional improvement in short-term follow-up, (D)Functional improvement in long-term follow-up.
